# Human rights versus societal norms: a mixed methods study among healthcare providers on social stigma related to adolescent abortion and contraceptive use in Kisumu, Kenya

**DOI:** 10.1136/bmjgh-2017-000608

**Published:** 2018-03-05

**Authors:** Miranda Håkansson, Monica Oguttu, Kristina Gemzell-Danielsson, Marlene Makenzius

**Affiliations:** 1 Department of Public Health Sciences/Global Health (IHCAR), Karolinska Institutet, Stockholm, Sweden; 2 Kisumu Medical and Education Trust (KMET), Kisumu, Kenya; 3 Department of Women’s and Children’s Health, Division of Obstetrics and Gynaecology, Karolinska Institutet, Karolinska University Hospital, Stockholm, Sweden

**Keywords:** stigma, abortion, contraception, adolescent pregnancy, healthcare providers

## Abstract

**Introduction:**

Adolescent pregnancy represents a serious public health issue in sub-Saharan Africa, and stigmatising attitudes are contributing factors. This study investigates stigmatising attitudes related to adolescent pregnancy, abortion and contraceptive use among healthcare providers working with postabortion care (PAC) in a low-resource setting in Kenya.

**Methods:**

A mixed methods approach in a convergent design was utilised to capture attitudes related to abortion and contraceptive use among 86 (f=62; m=19) PAC providers in Kisumu, Kenya. Two Likert-scale questionnaires were used: the 18-item Stigmatising Attitudes, Beliefs and Actions Scale (SABAS) and the 7-item Contraceptive Use Stigma Scale (CUSS). 74 PAC providers responded to the SABAS, 44 to the CUSS and 12 participated in two focus group discussions. Descriptive statistics, psychometric tests of instruments and qualitative content analysis were conducted and reported in accordance with Consolidated Criteria for Reporting Qualitative Research.

**Results:**

Cronbach’s α coefficients for the total instrument was 0.88 (SABAS) and 0.84 (CUSS). The majority, 92% (68/74) agreed that a woman who has had an abortion should be treated equally to everyone else, 27% (20/74) considered abortion a sin and 30% (22/74) believed she will make abortion a habit. Contraceptive use among adolescent women was associated with promiscuity (39%; 17/44), hence contraceptives should only be available to married women (36%; 16/44), and 20% (9/44) believed that contraceptive use causes infertility. The providers encouraged women’s autonomy and their rights to sexual and reproductive health; however, unclear regulations reinforce religious and cultural beliefs, which hampers implementation of evidence-based contraceptive counselling.

**Conclusion:**

Stigmatising attitudes towards young women in need of abortion and contraception is common among PAC providers. Their work is characterised by a conflict between human rights and societal norms, thus highlighting the need for interventions targeting PAC providers to reduce stigma and misconceptions related to abortion and contraception among adolescent women.

**Key questions**

**What is already known about this topic?**

- Adolescent pregnancy is a major public health issue in many low-income and middle-income countries that is associated with higher maternal mortality rates and sustained poverty in vulnerable groups.
- Women in need of abortion and contraception have reported that stigmatising attitudes among healthcare providers might create a significant barrier to safe sexual and reproductive health services, especially for adolescents.
- Understanding of the social stigma in healthcare settings is essential to mitigate the unmet need for contraception and prevent adolescent pregnancies; however, this objective is rarely investigated among healthcare professions.

**What are the new findings?**

- While providers of postabortion care (PAC) have a pragmatic view on adolescent autonomy regarding sexual and reproductive health, grounded in fundamental human rights, a general resistance to provide abortion and contraceptive services exists.
- PAC providers commonly believe that abortion is a sin, that once a woman has had one abortion she will make it a habit, and associate contraceptive use among adolescent women with a promiscuous lifestyle, hence thought that contraceptives should only be available to married women.
- The lack of clear regulations and policies on abortion in Kenya contributes to reinforce stigmatising attitudes based on religious and cultural values, which impedes PAC providers’ implementation of evidence-based contraceptive counselling.

**Key questions**

**Recommendations for policy**

- This study highlights the importance of continued implementation of youth-friendly clinics in Kenya addressing adolescents’ sexual and reproductive health needs and rights.
- The findings confirm that Kenya’s abortion policy must be clarified to healthcare providers and the public to ensure women have access to evidence-based contraception services, and to legal and safe abortion practices.
- Interventions targeting PAC providers and ethics discussions in medical pregraduation training are needed to reduce stigma and misconceptions on abortion and contraception.

## Introduction

Adolescent pregnancy and its consequences represent a major public health issue in many countries of the world. In low-income and middle-income regions, there were an estimated 21 million pregnancies among adolescent women aged 15–19 years in 2016. Nearly half (49%) of these pregnancies were unintended, and more than half of the unintended pregnancies ended in induced abortion.^1^

Young pregnant women have an increased risk of maternal morbidity and mortality, and a higher rate of induced and often unsafe abortions compared with women of other ages.^2–4^ Unsafe abortion causes 13% of all maternal deaths, making it one of the dominant causes of maternal mortality.^5^ Moreover, becoming pregnant at a young age often forces girls to leave school and consequently curtail their education, which results in sustained poverty and increased vulnerability.^6^ The United Nations Sustainable Development Goal three aims to reduce the global maternal mortality ratio to <70 per 100 000 live births by 2030. In 2015, the estimated ratio was 216 per 100 000 live births.^7^ The main underlying causes to death by unsafe abortion are no longer blood loss or infection, but rather that women’s health issues are rarely prioritised.^8–10^

Abortion stigma is widely acknowledged, and its sources may include controversies about reproductive physiology, normative sexuality, abortion laws and policies and religious norms and cultural beliefs. Fear of discrimination and social exclusion keep people from speaking out in support of those who abort, thus sustaining the negative stereotype.^11^ Although contraceptives have become more accessible and affordable during the last decades, misconceptions among the public and professional healthcare providers about the use of contraceptives continue to block adolescents from using effective contraceptive methods.^12 13^ While these misconceptions maintain the stigma related to contraception, the social constructs that cause this stigma can be deconstructed.^11^

There are currently 10.5 million adolescents aged 10–19 years living in Kenya, corresponding to 22.5% of the country’s total population. The national fertility rate is 3.9, and the contraceptive prevalence rate is estimated to be 43% among all women aged 15–49 years and 58% among married women. Predominantly used contraceptive methods among all women are injectable contraceptives (19%), hormonal implants (7%) and contraceptive pills (6%).^14^ According to a new constitution adopted in 2010, abortion may now be granted to a pregnant woman if her health is at risk, based on an evaluation performed by a trained health professional.^15^ However, as the majority of the induced abortions in Kenya are still unsafe, abortions remain a leading cause of maternal morbidity and mortality in the country; young women aged 10–19 years are the most vulnerable.^16^

Stigmatising attitudes among healthcare providers is one explanation to why pregnant women seek help outside healthcare facilities.^17^ Kenyan women describe abortion safety in terms of their physical health, and in terms of their social, reputational, relationship and economic security.^18^ These women therefore prefer abortion services that are secret, affordable and identified through trusted social networks, even though such services often are clandestine.^17 18^ However, an understanding of how the social stigma on abortion and contraceptive use is expressed among healthcare providers who provide abortion and contraceptive services is limited. Thus, understanding the healthcare providers’ views and attitudes is essential to mitigate the unmet need for contraception and prevent adolescent pregnancies. This study therefore explores stigmatising attitudes related to adolescent pregnancy, abortion and contraception among healthcare providers providing postabortion care (PAC) in a low-resource setting in Kisumu, Kenya.

## Materials and methods

### Study design

**Figure 1:**
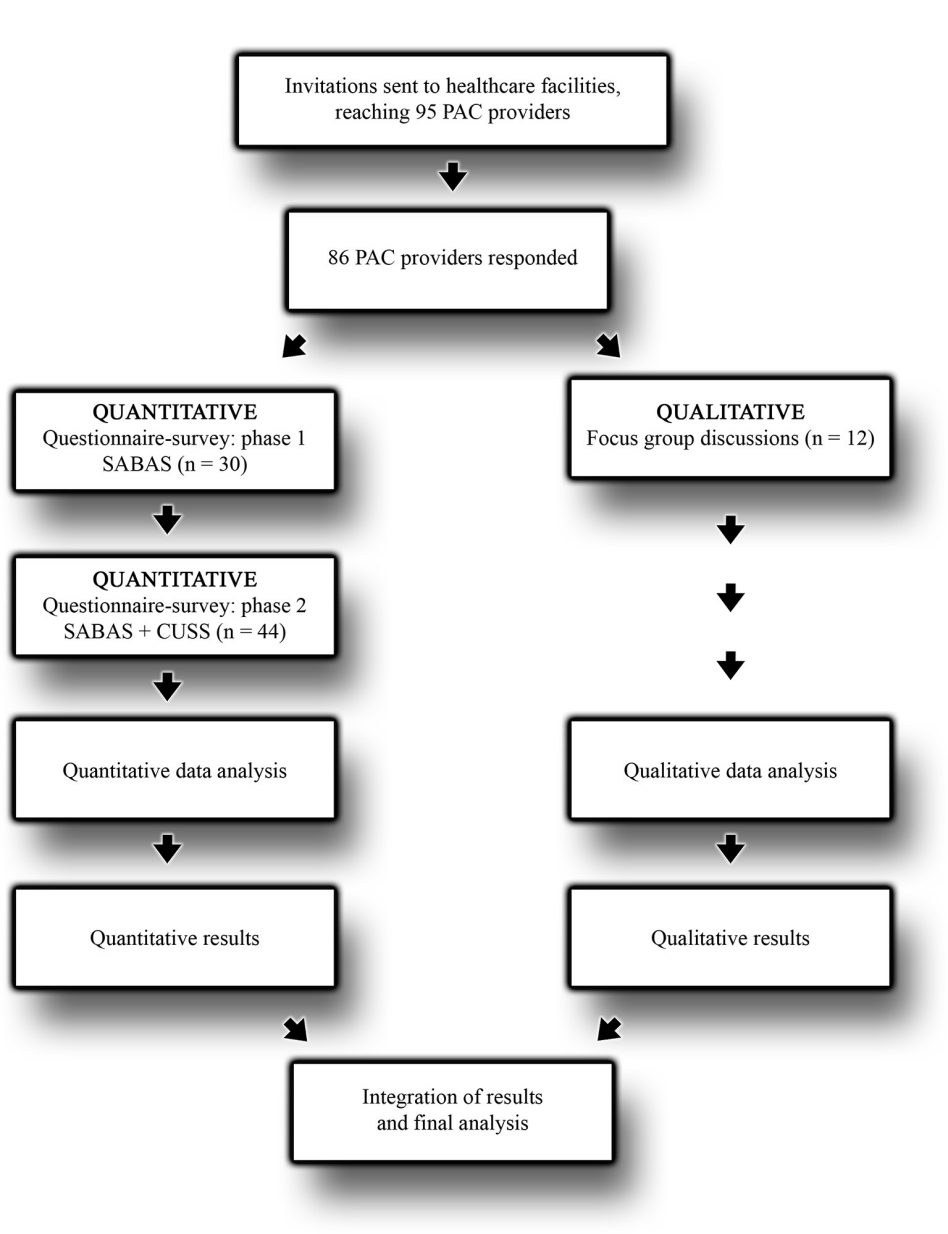
The data collection process illustrating the mixed methods approach in a convergent study design. CUSS, Contraceptive Use Stigma Scale; PAC, postabortion care; SABAS, Stigmatising Attitudes, Beliefs and Actions Scale.

This mixed methods study is a substudy nested in a cluster randomised intervention study, registered at ClinicalTrial.gov (NCT03065842;pre-results). The study uses a mixed methods approach in a convergent design according to Creswell *et al*.^19^ Quantitative and qualitative data were collected and analysed concurrently but separate, then the results from the two data sources were merged and integrated (figure 1). The purpose of using two different methods was to triangulate the quantitative and qualitative data to obtain a deeper understanding of the meaning and implications of the result.^20^ The study is based on two Likert-scale questionnaires and two focus group discussions (FGDs), to capture PAC providers’ attitudes towards abortion and contraceptive use among young women. The reporting was done in accordance with the guidelines for Strengthening the Reporting of Observational Studies in Epidemiology^21^ and the Consolidated Criteria for Reporting Qualitative Research.^22^

### Study setting

The study was conducted within Kisumu East District in Kisumu, western Kenya. Kisumu is the third largest city in Kenya, with a population of approximately 500 000. An estimated 60% of the population live in informal settlements and about 44% are younger than 15 years. The contraceptive prevalence rate is estimated to 27%, with the lowest rate of contraceptive use among sexually active women aged 15–19 years.^23^ Around 70% of the Kenyan population are Protestant, 20% are Roman Catholic and 7% are Muslim.^14^ Participants were recruited by purposive sampling from four healthcare facilities that provide PAC and youth friendly services within the study area. The included facilities were Kisumu County Hospital (KCH), Jaramogi Oginga Odinga Teaching and Referral Hospital (JOOTRH), Kisumu Medical and Education Trust (KMET) and one private Christian faith-based healthcare clinic. KCH and JOOTRH are the two main public hospitals in Kisumu. KMET is a non-religious non-governmental organisation (NGO), which has two healthcare clinics.

### Study participants

Invitations to participate voluntarily in the questionnaire-survey or the FGDs were sent to the included healthcare facilities. This was considered the most effective way to reach all providers working at the clinics, instead of trying to reach each specific possible participant separately. During the time of data collection, 95 healthcare providers experienced in PAC and youth friendly services worked at the included healthcare facilities. Professions included for the questionnaire-survey were nurses, midwives, physicians, clinical officers, pharmacists, social workers and NGO staff working with PAC and adolescents at the included clinics. Healthcare providers located within the study area but without experience of PAC and youth friendly services were excluded from the study.

To participate in the FGDs, the inclusion criteria were midwives or physicians experienced in PAC, because these professions are most heavily involved in medical decisions on abortion, contraceptive counselling and prescriptions in Kenya.

### Data collection

#### The questionnaire-survey

The Stigmatising Attitudes, Beliefs and Actions Scale (SABAS) was a newly validated tool designed in 2013 for measuring stigmatising attitudes and beliefs about abortion in Ghana and Zambia.^24^ The scale has the potential to be applicable in other country settings,^24^ and comprises three subscales: (1) negative stereotyping, (2) exclusion and discrimination and (3) fear of contagion, in total 18 items. The Contraceptive Use Stigma Scale (CUSS) was compiled within the study and contained seven items. The response categories were set up on a Likert-scale ranging from 1 (‘strongly disagree’) to 5 (‘strongly agree’).

The questionnaire-survey was carried out in two phases commencing with a pilot study of 30 PAC providers in November 2016. The PAC providers attended a workshop about PAC and completed the original SABAS anonymously on arrival at the workshop before receiving any other information aside from that given in the invitation letter. During the workshop, the items of the SABAS were discussed to determine the relevance of the scale for PAC providers in the context of Kisumu for further use within the study. As the SABAS was considered relevant without any adjustments, the SABAS was used in its original form for the questionnaire-survey. The CUSS was discussed, compiled and tested during the same workshop, and minor revisions were made based on four PAC providers’ evaluations.

In January 2017, the second quantitative data collection was performed. The SABAS and CUSS were distributed to the two included public hospitals, KCH and JOOTRH, and 44 PAC providers responded. Each participant was provided with the questionnaires and an envelope. A focal person at each facility collected the completed questionnaires in the sealed envelopes and delivered them to the research assistant within 4 days.

#### The FGDs

Two FGDs were conducted in a private setting at the collaborating organisation KMET during the workshop in November 2016. Twelve PAC providers, who did not attend the workshop or respond to the questionnaires, participated voluntarily in the discussions. One male and five female midwives and physicians participated in each focus group. One female senior professor and one female senior PhD researcher (MM), both experienced in qualitative methods within the objective, trained and supervised four fieldworkers (two females and two males) to act as moderators and notetakers in the two FGDs. The fieldworkers all had previous experience of PAC and leading focus groups. Two fieldworkers, one of each sex, attended each FGD, and none had any previous relationship to the participants. The background of the research project and the purpose of the discussions were explained to the participants prior to commencing the FGDs. A topic guide and open questions were used as the basis for the discussions to explore the participants’ views on unintended pregnancy, abortion and contraceptive use among adolescents. The discussions were held in English and each lasted around 75–90 min. A Sony ICD-PX312 digital voice recorder was used to record the discussions, which were transcribed verbatim within 5 days thereafter.

### Data analysis

#### The questionnaire-survey

All data from the questionnaires were entered into SPSS V.22.0 and then analysed by descriptive statistics. Shellenberg *et al*, the developers of the SABAS,^24^ conducted an exploratory factor analysis to identify a statistically and conceptually relevant scale. We repeated this analysis in our sample. Restricting to a three-factor solution, we found that 56% of the variance in a 17-item, rather than 18-item instrument. The three identified subscales were a mixture of Shellenberg *et al*^24^ negative stereotypes (all eight items), the discrimination and exclusion (six of the seven items found by Shellenberg) and one item from the potential contagion (which had three items in subscale of Shellenberg). We found a second component which is the potential contagion subscale of Shellenberg, and a third subscale that contained only two items from the second subscale for discrimination and exclusion. Items with factor loading <0.39 were excluded, and Kaiser-Meyer-Olkin test=0.76, and Bartlett test χ^2^=657, df=153 (P<0.001), which verify suitability of the data. Cronbach’s α coefficient was 0.88, confirming the internal consistency reliability.

Understanding the limits of our sample size, to verify suitability and the dimensions of the data, we undertook a principal component analysis of the CUSS, finding a single factor containing sex of the seven items (one item loaded >0.39), which explained 53% of the total variation and a Cronbach’s α of 0.84, confirmed the suitability of data and internal consistency reliability.

The scores for the SABAS and CUSS were dichotomised: scores of 3–5 (‘agree’) were considered stigmatising attitudes, and scores of 1–2 (‘disagree’) were considered non-stigmatising attitudes (table 2).

#### The FGDs

The transcripts from the FGDs were analysed using qualitative content analysis according to Graneheim and Lundman.^25^ The first author, a female medical student, listened to the audio recordings and transcripts several times to obtain a sense of the whole and to extract meaning units. The meaning units were condensed and each was labelled with a code. The codes were compared for differences and similarities and grouped into several subcategories and then into categories. The categories were refined through a process of reflection and discussion between the first and last authors, who both participated in all stages of the study. Finally, two themes that unified the content in the categories were composed using latent content analysis.^25^ Throughout the analysis, all findings were discussed thoroughly within the research group to improve the consistency and accuracy of the coding and the interpretation. Two participants of the FGDs validated the results.

#### Integration of the quantitative and qualitative results

After concurrent, yet separate and independent collection and analysis of data, the quantitative and the qualitative results were compared for convergence, divergence and relation. In a final step, the two sets of results were merged and integrated to one overall interpretation to achieve a deeper understanding of the study objectives.

### Ethics

All participants were informed about the aim of the study, that their participation was voluntary and that they could withdraw from participation at any time without consequences. All participants gave their written informed consent. No economic compensations were given to any participant.

### Role of the funding source

The authors designed this as an investigator-initiated study, in collaboration with healthcare providers and management at the study setting. The funders did not play any role in the study design or in the collection, analysis and interpretation of data. All authors had full access to the data, and the first author regularly reported preliminary results to the whole research team. The corresponding author had final responsibility for the decision to submit manuscript for publication.

## Results

In total, 86 PAC providers participated in the study (table 1). Out of 95 PAC providers that worked at the included healthcare facilities during the time of data collection, 74 responded to the SABAS, which corresponds to a response rate of 78%. For the CUSS, 44 out of 49 available PAC providers at the two public hospitals responded, corresponding to a response rate of 90%.

**Table 1:** Demographics of participants (n=86)

	n
Gender	
Female	62
Male	19
Not stated	5
Age	
Min	21
Max	54
Mean (estimated)	32
Healthcare facilities	
Public hospitals*	69
NGO healthcare clinics	14
Private faith-based clinic	3
Profession	
Nurse/midwife	52
Clinical officer	11
NGO staff†	6
Social worker	5
Physician	4
Pharmacist	1
Not stated	7
Work experience‡	
Min	4
Max	30
Mean (estimated)	17

*Kisumu County Hospital and Jaramogi Oginga Odinga Teaching and Referral Hospital.

†Non-medical staff working with PAC and adolescents at the two healthcare clinics of the participating NGO.

‡Years of work experience of youth friendly services.

NGO, non-governmental organisation; PAC, postabortion care.

### The questionnaire-survey

The results of the questionnaire-survey (table 2) show that most of the SABAS participants, 92% (68/74) stated that a woman who has had an abortion should be treated the same as everyone else, yet stigmatising attitudes were evident. Thirty-five per cent of the respondents (26/74) thought that women who abort might encourage other women to have abortion, 30% (22/74) believed that once a woman has had one abortion she will make it a habit and 27% (20/74) of the providers considered abortion a sin. Of the 44 CUSS participants, 39% (17/44) stated that a young girl using contraceptives will encourage other girls to lead a promiscuous lifestyle, 32% (14/44) considered a young girl using contraceptives as promiscuous, 36% (16/44) agreed that contraceptives should only be available to married women and 32% (14/44) considered an adolescent woman too young to decide whether she should use a contraceptive method. Twenty per cent (9/44) believed that a girl who uses contraceptives will have problem when she decides to get pregnant.

**Table 2:** The SABAS and CUSS

		M/Med/SD/range (score 1–5)	Agree (score 3–5) n (%)	Disagree (score 1–2) n (%)
Items: SABAS				
1	The health of a woman who has an abortion is never as good as it was before the abortion (n=74).	2.3/2/1.2/4	26 (35)	48 (65)
2	A woman who has had an abortion might encourage other women to get abortions (n=74).	2.4/2/1.3/4	26 (35)	48 (65)
3	Once a woman has one abortion, she will make it a habit (n=74).	2.2/2/1.2/4	22 (30)	52 (70)
4	A woman who has an abortion is committing a sin (n=74).	2.2/2/1.4/4	20 (27)	54 (73)
5	A woman who has an abortion brings shame to her family (n=74).	2/2/1.1/4	16 (22)	58 (78)
6	A woman who has had an abortion brings shame to her community (n=74).	1.9/2/1.1/4	13 (18)	61 (82)
7	A woman who has had an abortion cannot be trusted (n=74).	1.7/1/1/4	12 (16)	62 (84)
8	A woman who has had an abortion is a bad mother (n=73).	1.6/1/.9/4	7 (10)	66 (90)
9	A woman who has an abortion should be treated the same as everyone else* (n=74).	1.2/1/.5/3	68 (92)	6 (8)
10	A man should not marry a woman who has had an abortion because she may not be able to bear children (n=74).	1.5/1/.8/4	5 (7)	69 (93)
11	I would tease a woman who has had an abortion so that she will be ashamed of her decision (n=74).	1.4/1/.7/4	4 (5)	70 (95)
12	I would try to disgrace a woman in my community if I found out she would had an abortion (n=74).	1.6/1/.9/4	4 (5)	70 (95)
13	I would stop being friends with someone if I found out that she had had an abortion (n=73).	1.4/1/.7/4	3 (4)	70 (96)
14	A woman who has had an abortion should be prohibited from going to religious services (n=74).	1.2/1/.5/2	2 (3)	72 (97)
15	I would point my fingers at a woman who had an abortion so that other people would know what she has done (n=74).	1.3/1/.5/2	1 (1)	73 (99)
16	If a man has sex with a woman who has had an abortion, he will become infected with a disease (n=74).	1.4/1/.8/4	7 (9)	67 (91)
17	A woman who has an abortion can make other people fall ill or get sick (n=74).	1.4/1/.8/4	6 (8)	68 (92)
18	A woman who has an abortion should be isolated from other people in the community for at least 1 month after having an abortion (n=74).	1.3/1/.8/4	4 (5)	70 (95)
Items: CUSS				
19	A young girl who uses a contraceptive method will encourage other girls to lead a promiscuous lifestyle (n=44).	2.4/2/1.3/4	17 (39)	27 (61)
20	A married woman is more deserving of a contraceptive method than an unmarried woman (n=44).	2.4/2/1.5/4	16 (36)	28 (64)
21	A young girl who uses a contraceptive method is promiscuous (sexually immoral, likes to have many sexual relationships) (n=44).	2.2/2/1.2/4	14 (32)	30 (68)
22	A young girl cannot decide for herself whether to use a contraceptive method (n=44).	2.4/2/1.3/4	14 (32)	30 (68)
23	A young girl who carries condoms is likely to have many sexual partners (n=44).	2.1/2/1.3/4	12 (27)	32 (73)
24	A young girl who uses contraceptives will have problems when she decides to get pregnant (n=44).	1.9/2/.9/3	9 (20)	35 (80)
25	A young girl should not insist on using a condom; the man should decide whether to use a condom or not. (n=44)	1.4/1/.9/4	3 (7)	41 (93)

Stigmatising attitudes towards women associated with abortion and contraceptive use among PAC providers (n=74; n=44) in a low-resource setting in Kenya.

*This item was reversed coded so that ‘agree’=1–2 and ‘disagree’=3–5; accordingly, ‘agree’ in this item was considered a non-stigmatising attitude.

CUSS, Contraceptive Use Stigma Scale; PAC, postabortion care; SABAS, Stigmatising Attitudes, Beliefs and Actions Scale.

When given the option to leave a comment, two providers stated that abortion should be completely illegal, and five commented that it should be legal only when the mother’s or the baby’s life is at risk. One provider stated: *‘Abortion is preferable in the case of saving the life of the mother, but not to escape shame or responsibility’.*

### The FGDs

The qualitative content analysis identified 22 subcategories, 8 categories and 2 themes—*human rights* and *societal norms—*which are presented in tables 3 and 4.

**Table 3:** The theme human rights*—* illustrated by categories, subcategories and selected codes emerged from two FGDs with PAC providers (n=12) in a low-resource setting in Kenya

Theme	Category	Subcategory	Code
Human rights	Acceptance	Abortion exists in our society	We must accept that abortion is common in Kenya.
			Induced abortions occur daily.
			Most unintended pregnancies will never reach term.
		Her decision	Abortion should be the choice of the woman.
			If she wants to terminate she will do it either way.
	Economical aspects	Recognising personal losses	Abortion services are too expensive.
			Unsafe abortions can incur expensive costs for PAC.
			Unsafe abortions cause death and loss of family members.
		Safe abortion to reduce societal costs	Providing safe abortions would be cheaper than providing PAC.
			PAC is financed by taking resources from other areas within healthcare.
			Orphaned children become a burden on society.
	Prevention	Importance of counselling	Counselling is essential.
			Proper counselling can prevent psychological trauma.
			Counselling can help the client make informed choices.
		Availability	Youths should be received in a good atmosphere.
			Her case must always be taken seriously.
			Follow-ups are important.
		Prevent ignorance and misconceptions	Find out the myths and misconceptions.
			Explain possible side effects.
			Provide sexual and reproductive health education to all.
		Benefits of contraceptives	Prevent girls from dropping out of school.
			Give girls a chance to create their future.
			Enable birth spacing within families.
	Gender equality	Increase male involvement	Male involvement brings peace in the home.
			Address the persons responsible for the pregnancies.
			Most of the policymakers are men.
			Easier to access women through their husbands.
		Empowerment of young women	Empower girls to negotiate for safer sex.
			Strengthen girls’ confidence in relationships.

FGD, focus group discussions; PAC, postabortion care.

**Table 4:** The theme societal norms—illustrated by categories, subcategories and selected codes emerged from two FGDs with PAC providers (n=12) in a low-resource setting in Kenya

Theme	Category	Subcategory	Code
Societal norms	Moral and religious beliefs	Moral judging	You are still too young to be sexually active.
			Using contraceptives means you are a bad girl.
			A woman without children should be a virgin.
			Everyone thinks abortion is shameful.
		Religious, cultural and legal crime	Procuring an abortion is like killing.
			You have committed a sin.
			Abortion is criminal.
		Personal values	Providers are the same people you meet in church.
			You can easily counsel a girl out of the abortion.
			It is not necessary to terminate that pregnancy.
			A young person should not be given contraceptives.
		Abortion requires valid indications	When the mother is at risk.
			When there is gross fetal malformation.
			In cases of rape.
	Myths and misconceptions	Believed side effects of contraceptives	Infertility.
			Giving birth to abnormal children.
			Long-acting reversible contraceptives cannot be provided to young women without children.
		Contraceptive use leads to promiscuity	A girl given access to contraceptives will start sleeping around.
			She is going to become a dropout.
	Societal resistance	Lack of a clear abortion policy	A clear policy on abortion is needed.
			Everyone still believes abortion is a crime.
			Others will view you as a criminal.
		Fear of prosecution	We were told to withdraw the term ‘ safe abortion ‘.
			If you do abort, the law will catch up with you.
			Our hands are tied.
		Institutional blocks	The policymakers stop us from talking about abortion.
			Teachers are not allowed to talk about abortion and contraceptives.
			Focus on advocacy through the church leaders.
	Women’s inferiority	Lack of autonomy	Young girls have intergenerational sex for money.
			Men do not take responsibility for the pregnancies.
			Men make decisions for their women.
			Her parents will stop paying her school fees.
		The burden of secrecy	Girls have abortions secretly to hide it from their parents.
			An unintended pregnancy lowers her status in the community.
			She cannot share her secret with anyone.
		Exclusion and discrimination	A pregnant girl may be considered an outcast.
			People do not want to be associated with her.
			She will be isolated.
			That girl using contraceptives will be labelled a sex worker.
			She will be mistreated by parents, teachers and the community.

FGD, focus group discussions; PAC, postabortion care.

### Human rights

The theme *human rights* emerged from four categories: acceptance, economical aspects, prevention and gender equality (table 3). This theme illustrates how the PAC providers strive to accept young women’s sexual and reproductive health needs and issues to prevent adolescent pregnancy and defend women’s human rights.

#### Acceptance

Most of the PAC providers expressed a pragmatic view on adolescent pregnancy, abortion and contraceptive use referring to fundamental human rights. Even though most providers preferred young people to abstain from sexual activities, they acknowledged that abstinence was not always possible. The providers agreed that unintended pregnancies would most likely be terminated one way or another. Furthermore, there was a common awareness that abortion procedures are frequent in Kenya and that all PAC providers must deal with them in their daily work. Some participants claimed that no specific indications for abortion should be needed since it should be a woman’s right to make independent decisions about her body.

Once she has decided, ‘I don’t want to keep this pregnancy’, it doesn’t matter how much counselling you are going to give this woman. Eventually she will terminate the pregnancy and she doesn’t care where […], she will get it the unsafe way. (Participant 12)

#### Economical aspects

While some participants mentioned that women often choose unsafe procedures because they cannot afford to pay for a safe abortion, other participants claimed that unsafe abortions could be even more expensive because of the high risk of complications and the need for costly PAC services. The loss of income to families or even the loss of family members was also mentioned. Unsafe abortions were also considered a burden on the public healthcare system because resources are taken from other areas to finance PAC. Providing safe abortion services within public healthcare would thus be a more cost-effective option:

Our facilities would not have the expense of using a lot of other resources to help these clients because it is cheaper when it is done safely than when it is done after things have been messed up. (Participant 11)

#### Prevention

Several participants emphasised the importance of counselling to decrease psychological stress and to guide the youths through their choices and decisions. They believed that providers should give correct information and act as non-judgemental counsellors rather than decision makers:

But actually, we are only there to counsel and then the client should make an informed choice. (Participant 7)

Youth-friendly services were considered important for contraceptive counselling and abortion services. Adolescents already have many hurdles to overcome before they can even make the decision to seek help at a healthcare facility, and they should therefore feel welcome and be taken seriously once they arrive at the clinic. Nevertheless, the occurrence of well-reasoned counselling was said to be rare, either due to a lack of time or a provider’s resistance to providing the required services. The participants therefore welcomed the concept of youth-friendly clinics (YFCs) that offer liberal opening hours, have adequate equipment and supplies and are visible to all in the community. Comprehensive sex education was considered essential to prevent ignorance and misconceptions about contraception and abortion, and to increase compliance with contraceptive methods.

In fact, even before you start counselling a client on a method, first you need to say, ‘tell me about the myths and misconceptions you have about this method’. They will tell you so many things. […], when they get any condition, they relate it to family planning. (Participant 4)

Several benefits of contraceptives were mentioned such as preventing young girls from dropping out of school due to unplanned pregnancies and the related stigma, and enabling birth spacing within marriage.

#### Gender equality

The substantial gender power imbalance was considered a root cause of young girls’ vulnerability in society. As most policymakers in Kenya are men, some participants stated that for any changes to happen, these men need to be more involved. Participants recognised that it can be difficult to reach out to women without also involving their husbands, adding that young women need to be empowered to strengthen their confidence in relationships. Women need tools to negotiate for safer sex; however, male involvement was considered essential to decrease gender inequality.

Men always feel that they are the decision makers and that they should be consulted before something is done, just to make them feel that they actually own this woman. (Participant 2)

We can empower young girls on the use of contraceptives, but we also have to bring about the responsible persons for the pregnancies, who are the men and the young boys. (Participant 10)

### Societal norms

The theme *societal norms* emerged from four categories: moral and religious beliefs, myths and misconceptions, societal resistance and women’s inferiority (table 4). This theme illustrates how religious and cultural values moralise sexuality, abortion and contraceptive use among young women. This theme also shows the consequences of these values when policies are unclear.

#### Moral and religious beliefs

Although most providers expressed pragmatic opinions on adolescent pregnancy, the general view was that neither abortion nor contraceptive use among young people are accepted in sub-Saharan African societies. Adolescent sexual activities were described as immoral and irresponsible behaviour. Losing virginity before entering marriage was considered a disgrace, and participants explained that few men would marry women who have already given birth. Instead, to avoid bringing shame on themselves or their families, the young and unmarried were expected to practice strict self-restraint, since virginity was highly valued.

Induced abortion was described not only as a legal crime, but also as a religious and cultural taboo. Some providers equated abortion with killing, and thus considered it a sin. If a woman considered abortion an option, they suggested that proper counselling could rectify the woman’s decision and make her keep the pregnancy. Abortion could only be justified in accordance with the law when the mother’s health is at risk. However, the interpretations of the kind of health risks that were lawful were not clearly specified. Other acceptable motives were in cases of fetal malformation or after rape. However, it was clear that abortion should not be done merely on a woman’s request:

Another case is if a mother comes to me saying, ‘please help me, my target was three children and I have realised I have a fourth one, but I don’t want this fourth one’. Such cases should be counselled well and should not be allowed to have the abortion. (Participant 8)

#### Myths and misconceptions

Contraceptive use was related to several physical conditions of varying severity, ranging from weight gain or weight loss, irregular bleedings and loss of libido to different types of malignancies, fetal malformations and infertility. Long-acting reversible contraception (LARC) was considered particularly questionable for young women without previous pregnancies. Some participants expressed concern about an increased risk of persistent infertility after LARC use and were afraid of being blamed for causing infertility due to their counselling and provision of contraception. The participants therefore preferred young people to practice abstinence from sexual activities and stressed the importance of counselling to encourage such abstinence. The participants explained that society’s normative disapproval of contraception should be respected and that no form of contraception should be provided to young unmarried women. Some of the participants expressed that access to contraception and sex education could encourage promiscuity and irresponsible behaviour, which was referred to by a provider as an *‘unhealthy lifestyle’*:

If you are using contraceptives, then maybe you are a sex worker, you are a bad girl, and you sleep around. That’s why you are using contraceptives, you don’t take care of yourself. (Participant 2)

#### Societal resistance

Uncertainties on how to interpret the meaning of the Kenyan abortion law caused problems for the PAC providers. Regardless of the participants’ views of the current law on abortion, all wanted clearer policies on abortion and contraception services. Some considered it inconsistent for healthcare facilities to be allowed to provide PAC, but not to perform safe abortions. It was also discussed that many people in society, including some of the PAC providers, are unaware of the abortion law and still believe that abortion is completely illegal irrespective of the circumstances. Many participants expressed a fear of being prosecuted by law if they were to perform an abortion and therefore consistently choose to refuse to provide abortion services.

I wish I had known I could have offered the service, if I offered it safely then this mother could still be living [….] If there was a clear policy on abortion, a client can just come even to any public facility and get the service. This issue of giving tablets in the backstreet will just go away forever. (Participant 11)

Participants described that policymakers, teachers and church leaders persistently prevent PAC providers from talking about abortion and contraceptive use in the community, in the schools and in the churches. Some participants considered this problem as one of the main barriers to the preventive work and were frustrated because of their inability to help women with unintended pregnancies:

When they want to terminate the pregnancy, we just counsel them, the mothers and the young girls, because with the Kenyan law, abortion is not allowed. (Participant 1)

#### Women’s inferiority

The participants gave several examples of young women’s lack of autonomy. Sexual relationships between young girls and older men, called intergenerational sex, were described as a way for girls to get money to pay their school fees or buy things. These young girls are particularly vulnerable since their state of dependency limits their opportunities to negotiate safe sex. However, this problem also applied to women within marriage because many women need consent and economical support from their husbands to gain access to contraception. The man was often referred to as *‘the owner of the pregnancy’.*

The social stigma surrounding unintended pregnancies was considered one of the most important reasons why many young women try to induce abortion themselves or with the help of a quack (a fraudulent person, lacking scientific medical knowledge and/or skills).

A man just wanted to have a good time, but unfortunately a pregnancy results. The man just disappears and this girl is left with no choice but an unsafe abortion. (Participant 5)

The providers explained that pregnant girls are banned from their schools, and most providers had met girls who had been sent home from school after their teachers had palpated the students’ arms at the school gates and found an implant. The participants considered this situation problematic since most providers agreed that the implant was one of the better LARC options for young girls in need of contraception. The participants also referred to some communities where a woman who has had an abortion would be kept isolated and would not be allowed to be close to pregnant women or small children for at least 4 weeks.

Their teachers always associate these contraceptives, like if you are on one, they view you as spoiled, immoral, that you just love men, and you don’t concentrate on your studies. (Participant 3)

### The controversy in the prevention of adolescent pregnancy

**Figure 2:**
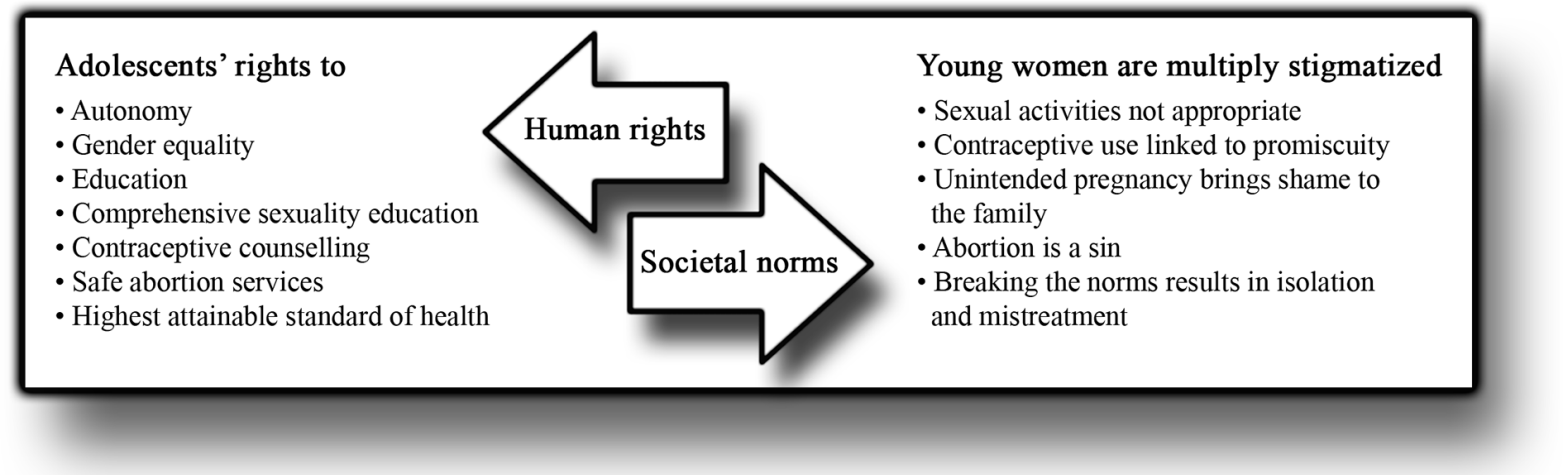
A model of the controversy in preventing adolescent pregnancy—the conflict between fundamental human rights and societal norms.

Figure 2 presents the integration of the results from the quantitative and qualitative data analyses. This summarising figure reflects the controversy involved in preventing adolescent pregnancy, characterised by a conflict between fundamental human rights and societal norms. This is a complex conflict that involves healthcare providers’ professional skills and personal attitudes, as well as societal norms in this particular context. This stress the importance of implementing locally contextualised interventions that build on understanding of family formation, views on reproductive rights and on how the latter is being affected by the societal norms. The findings collected from the PAC providers in this study indicate that views on adolescent pregnancy and its prevention is currently changing in this setting. Interpretation of the model in figure 2 suggests that interventions designed to address adolescents’ sexual and reproductive health issues should meet the providers where they are in terms of context and baseline views.

## Discussion

The aim of this study was to explore stigmatising attitudes related to adolescent pregnancy, abortion and contraception among PAC providers in a low-resource setting. The results show that PAC providers’ views are characterised by a conflict between *human rights* and *societal norms.* The providers expressed a pragmatic view on adolescents’ human rights, such as the rights to life, autonomy, equality, education and the highest attainable standard of healthcare. The participants also communicated a general resistance to provide abortion services and contraceptive counselling, mainly reflecting societal norms such that adolescent sexual activities and thus contraceptive use and abortion are immoral and irresponsible.

An important advantage of the study design was the mixed methods approach which allowed triangulation. This technique facilitates validation and reliability of the data through cross-verification of the quantitative and qualitative data sources. Moreover, triangulation helps to obtain a deeper and more complete understanding of the studied phenomenon, particularly in sensitive objectives.^26^ A limitation of this study was the small sample size for the questionnaire-survey, which did not allow comparisons between subgroups of the sample. Given the small sample size, the CUSS was only qualitatively validated; however, because statistics confirmed the suitability of the data and internal consistency reliability of the scale, we provided summaries of the individual item responses and overall qualitative findings. A larger study by which to validate the scale is in progress within this research project, and this will allow us to use this scale quantitatively to test for differences in the future. Considering the sensitive nature of this study, the main purpose of the questionnaires was to explore if and how abortion and contraceptive use stigma is articulated among PAC providers. The dichotomisation grouped scores 3–5 as stigmatising attitudes and scores 1–2 as non-stigmatising attitudes. Score 3 stands for ‘unsure’, which means that a provider choosing this score did not take a stand against the given statement. Therefore, score 3 was considered a stigmatising attitude. By the mixed methods design, qualitative results could confirm the findings in the questionnaires and support the reliability of these results.

Owing to the limited time and financial resources, we conducted only two FGDs with only physicians and midwives. Nevertheless, the prior qualitative validation of the scales and the quantitative questionnaires supported to monitor and achieve data saturation. Trustworthiness of qualitative research is based on the concepts of credibility, dependability and transferability.^25^ Credibility was gained by the purposive sampling, which generated a heterogeneity within the sample in terms of age, sex, occupation and work experience. Bringing together a diverse group of people was considered advantageous to maximise the exploration of different perspectives in the group. However, the gender and hierarchy mix within the FGDs must be considered in this context since it might have affected the outcome of the discussions. Voluntary participation is a possible weakness because the participants might have stronger opinions, either positive or negative, compared with the average population.^27^ Nevertheless, FGDs have shown to facilitate such discussions since sharing the same experience might contribute to a deeper discussion from different perspectives.^27^ Most important, the goal was not to generalise the findings, but rather to indicate transferability through consistency with other studies conducted in various contexts.^12 13 17 28 29^ The consistency and accuracy were further strengthened by the heterogenic research team, with personnel experienced in quantitative and qualitative research within this objective and context. The dependability was fortified by conducting the two FGDs simultaneously to avoid participants interacting with each other in between the FGDs.

Worldwide, providers of sexual and reproductive health services are dealing with controversial issues such as sexuality, abortion and contraception.^30^ This study sheds light on the conflict between adolescents’ human rights against the norms and beliefs in society. Most providers in this study opined that a woman who has had an abortion should be treated equally to everyone else, but they still had negative stereotypes about women who abort. Furthermore, it is important to note that these providers are currently offering PAC services and have been trained in youth-friendliness, hence are likely to have more liberal attitudes than other healthcare providers.

In Kenya, as in many other contexts, women who seek to terminate a pregnancy challenge the ideals of womanhood that originate in conservative gender roles and intend to control female sexuality and compulsory motherhood.^11^ In the FGDs, the providers indicated young women’s lack of autonomy as one of the greatest challenges to provide sexual and reproductive health services to young women. Three out of 10 PAC providers considered an adolescent woman too young to decide whether to use contraception, a finding in accordance with previous studies of East Africa.^13 28^ Many of the PAC providers accentuated increased male awareness and involvement as an important measure towards improved sexual and reproductive health for women.

Participants of the FGDs consistently used terms such as sin, immorality and promiscuity, which reflected their society’s religious values and cultural beliefs. Young women seem to be multiply stigmatised when seeking abortion or contraceptive counselling, since having sex at a young age, having an abortion and continuing a pregnancy are equally unacceptable in the community. Previous studies have also shown that providers have social-based and moral-based restrictions regarding abortion and contraception, especially for young unmarried women.^12 17^ One explanation for these restrictions could be that, as the providers display themselves as a part of the community, they are also carriers of the existing norms. Religion is identified as one of the most important factors influencing providers’ attitudes and actions.^17^ Consequently, when policies are unclear, religious norms rather than scientific evidence seem to dictate the guidelines. In Kenya, an estimated 40% of the national health services are provided by faith-based organisations, most of them Christian, and many of those work in partnership agreements with the Ministry of Health.^31^ Providers in faith-based organisations might tend to focus contraceptive counselling on recommendations to abstain, even though promoting abstinence is not proven to delay initiation of sexual intercourse or decrease unintended pregnancies.^32^ Negative health effects have been noted when religious values are mixed with national policies, most strongly documented in relation to sexual and reproductive health.^30^

The providers were aware that the myths and misconceptions surrounding adolescent sexuality, abortion and contraception might keep young women from seeking abortion services and contraceptive counselling. This awareness highlights that PAC providers are unprepared to deal with the ambivalence when facing adolescents with sexual and reproductive health problems. Ethical dilemmas related to sexual and reproductive health and rights, as well as evidence-based education to prevent ignorance that might generate stigmatising attitudes, are important endeavours in medical pregraduation training.^33^

The interpretation of the slightly liberalised Kenyan abortion law adopted in 2010 was a hot topic during the FGDs, and the providers considered the vagueness of valid indications for abortion as a severe weakness in the new constitution. However, a liberal abortion law alone may not ensure the safety of abortions. Knowledge about the law must be disseminated, and abortion policies and guidelines need to be explicit. In addition, providers must be willing to obtain training and provide quality and non-judgemental abortion and contraceptive services, as systematically provided contraceptive counselling in PAC is effective to mitigate unmet need for contraception.^34^ Additionally, the governments must be committed to providing the necessary resources to ensure women can access those safe services, even in remote areas,^5^ and YFCs were considered an important resource for adolescents.

For future research, the SABAS and CUSS could be used in a larger sample of healthcare providers in similar settings to validate the relevance and the reliability of these scales. Since our findings indicate that stigmatising attitudes and misconceptions surrounding abortion and contraceptive use among adolescents are common in this community, such study could also provide a baseline for a stigma reduction intervention among PAC providers. Future research should also include adolescents in similar studies to explore their attitudes towards abortion and contraceptive use.

## Conclusions

Although most of the PAC providers stated that a woman who has had an abortion should be treated equally to everyone else, many still considered her sinful and promiscuous and thought she would make it a habit. In their role as healthcare professionals, PAC providers want to encourage adolescent’s rights to sexual and reproductive health; however, the lack of a clear abortion policy creates a gap that is guided by religious values and cultural beliefs and they therefore struggle between defending fundamental *human rights* and the *societal norms*. This struggle highlights the need for interventions targeting authorities on a policymaking level and PAC providers to reduce stigma surrounding abortion and contraceptive use, to mitigate the unmet need for contraception and prevent adolescent pregnancies.

## References

[1] Darroch JE, Woog V, Bankole A, et al Adding it up: costs and benefits of meeting the contraceptive needs of adolescents. New York: Guttmacher Institute, 2016.

[2] Nove A, Matthews Z, Neal S, et al Maternal mortality in adolescents compared with women of other ages: evidence from 144 countries. Lancet Glob Health 2014;2:e155–64. 10.1016/S2214-109X(13)70179-7 25102848

[3] Ganchimeg T, Ota E, Morisaki N, et al Pregnancy and childbirth outcomes among adolescent mothers: a World Health Organization multicountry study. BJOG 2014;121(Suppl 1):40–8. 10.1111/1471-0528.12630 24641534

[4] Shah IH, Ahman E Unsafe abortion differentials in 2008 by age and developing country region: high burden among young women. Reprod Health Matters 2012;20:169–73. 10.1016/S0968-8080(12)39598-0 22789095

[5] Sedgh G, Singh S, Shah IH, et al Induced abortion: incidence and trends worldwide from 1995 to 2008. Lancet 2012;379:625–32. 10.1016/S0140-6736(11)61786-8 22264435

[6] Neal SE, Chandra-Mouli V, Chou D Adolescent first births in East Africa: disaggregating characteristics, trends and determinants. Reprod Health 2015;12:13 10.1186/1742-4755-12-13 25971731 PMC4429933

[7] United Nations. Sustainable Development Knowledge Platform: Sustainable Development Goal 3. 2016 https://sustainabledevelopment.un.org/sdg3 (accessed 25 Feb 2017).

[8] Grimes DA, Benson J, Singh S, et al Unsafe abortion: the preventable pandemic. Lancet 2006;368:1908–19. 10.1016/S0140-6736(06)69481-6 17126724

[9] Ganatra B, Gerdts C, Rossier C, et al Global, regional, and subregional classification of abortions by safety, 2010-14: estimates from a Bayesian hierarchical model. Lancet 2017;390:2372–81. 10.1016/S0140-6736(17)31794-4 28964589 PMC5711001

[10] Gemzell-Danielsson K, Cleeve A Estimating abortion safety: advancements and challenges. Lancet 2017;390:2333–4. 10.1016/S0140-6736(17)32135-9 28964590

[11] Kumar A, Hessini L, Mitchell EM Conceptualising abortion stigma. Cult Health Sex 2009;11:625–39. 10.1080/13691050902842741 19437175

[12] Chandra-Mouli V, McCarraher DR, Phillips SJ, et al Contraception for adolescents in low and middle income countries: needs, barriers, and access. Reprod Health 2014;11:1 10.1186/1742-4755-11-1 24383405 PMC3882494

[13] Nalwadda G, Mirembe F, Tumwesigye NM, et al Constraints and prospects for contraceptive service provision to young people in Uganda: providers’ perspectives. BMC Health Serv Res 2011;11:220 10.1186/1472-6963-11-220 21923927 PMC3181204

[14] Kenya National Bureau of Statistics. Kenya Demographic and Health Survey 2014. 2014 https://dhsprogram.com/pubs/pdf/fr308/fr308.pdf (accessed 29 Jan 2017).

[15] National Council for Law Reporting. The Constitution of Kenya 2010. 2010 http://www.icla.up.ac.za/images/constitutions/kenya_constitution.pdf.

[16] Ziraba AK, Izugbara C, Levandowski BA, et al Unsafe abortion in Kenya: a cross-sectional study of abortion complication severity and associated factors. BMC Pregnancy Childbirth 2015;15:34 10.1186/s12884-015-0459-6 25884662 PMC4338617

[17] Rehnström Loi U, Gemzell-Danielsson K, Faxelid E, et al Health care providers’ perceptions of and attitudes towards induced abortions in sub-Saharan Africa and Southeast Asia: a systematic literature review of qualitative and quantitative data. BMC Public Health 2015;15:139 10.1186/s12889-015-1502-2 25886459 PMC4335425

[18] Izugbara CO, Egesa C, Okelo R ‘High profile health facilities can add to your trouble’: Women, stigma and un/safe abortion in Kenya. Soc Sci Med 2015;141:9–18. 10.1016/j.socscimed.2015.07.019 26233296

[19] Creswell JW, Plano Clark VL Designing and conducting mixed methods research. 3rd edn London: Sage, 2017.

[20] Malterud K Qualitative research: standards, challenges, and guidelines. Lancet 2001;358:483–8. 10.1016/S0140-6736(01)05627-6 11513933

[21] von Elm E, Altman DG, Egger M, et al The Strengthening the Reporting of Observational Studies in Epidemiology (STROBE) statement: guidelines for reporting observational studies. Lancet 2007;370:1453–7. 10.1016/S0140-6736(07)61602-X 18064739

[22] Tong A, Sainsbury P, Craig J Consolidated criteria for reporting qualitative research (COREQ): a 32-item checklist for interviews and focus groups. Int J Qual Health Care 2007;19:349–57. 10.1093/intqhc/mzm042 17872937

[23] The County Government of Kisumu. Kisumu County: first county integrated development plan 2013-2017. 2013 https://kisumu.go.ke/download/3 (accessed 2 Dec 2017).

[24] Shellenberg KM, Hessini L, Levandowski BA Developing a scale to measure stigmatizing attitudes and beliefs about women who have abortions: results from Ghana and Zambia. Women Health 2014;54:599–616. 10.1080/03630242.2014.919982 25074064

[25] Graneheim UH, Lundman B Qualitative content analysis in nursing research: concepts, procedures and measures to achieve trustworthiness. Nurse Educ Today 2004;24:105–12. 10.1016/j.nedt.2003.10.001 14769454

[26] Östlund U, Kidd L, Wengström Y, et al Combining qualitative and quantitative research within mixed method research designs: a methodological review. Int J Nurs Stud 2011;48:369–83. 10.1016/j.ijnurstu.2010.10.005 21084086 PMC7094322

[27] Kitzinger J Qualitative research. Introducing focus groups. BMJ 1995;311:299–302. 10.1136/bmj.311.7000.299 7633241 PMC2550365

[28] Tumlinson K, Okigbo CC, Speizer IS Provider barriers to family planning access in urban Kenya. Contraception 2015;92:143–51. 10.1016/j.contraception.2015.04.002 25869629 PMC4506861

[29] Makenzius M, Tydén T, Darj E, et al Autonomy and dependence--experiences of home abortion, contraception and prevention. Scand J Caring Sci 2013;27:569–79. 10.1111/j.1471-6712.2012.01068.x 22913927

[30] Tomkins A, Duff J, Fitzgibbon A, et al Controversies in faith and health care. Lancet 2015;386:1776–85. 10.1016/S0140-6736(15)60252-5 26159392

[31] Olivier J, Tsimpo C, Gemignani R, et al Understanding the roles of faith-based health-care providers in Africa: review of the evidence with a focus on magnitude, reach, cost, and satisfaction. Lancet 2015;386:1765–75. 10.1016/S0140-6736(15)60251-3 26159398

[32] Oringanje C, Meremikwu MM, Eko H, et al Interventions for preventing unintended pregnancies among adolescents. Cochrane Database Syst Rev 2016;2:CD005215 10.1002/14651858.CD005215.pub3 26839116 PMC8730506

[33] Klingberg-Allvin M, Van Tam V, Nga NT, et al Ethics of justice and ethics of care. Values and attitudes among midwifery students on adolescent sexuality and abortion in Vietnam and their implications for midwifery education: a survey by questionnaire and interview. Int J Nurs Stud 2007;44:37–46. 10.1016/j.ijnurstu.2005.11.018 16413553

[34] Makenzius M, Oguttu M, Klingberg-Allvin M, et al Post-abortion care with misoprostol - equally effective, safe and accepted when administered by midwives compared to physicians: a randomised controlled equivalence trial in a low-resource setting in Kenya. BMJ Open 2017;7:e016157 10.1136/bmjopen-2017-016157 PMC5652492 29018067

